# The Effect of a Dairy-Based Recovery Beverage on Post-Exercise Appetite and Energy Intake in Active Females

**DOI:** 10.3390/nu8060355

**Published:** 2016-06-08

**Authors:** Meghan A. Brown, Benjamin P. Green, Lewis J. James, Emma J. Stevenson, Penny L. S. Rumbold

**Affiliations:** 1 Department of Sport, Exercise and Rehabilitation, Faculty of Health and Life Sciences, Northumbria University, Northumberland Building, Newcastle upon Tyne NE1 8ST, UK; meghan.brown@northumbria.ac.uk (M.A.B.); penny.rumbold@northumbria.ac.uk (P.L.S.R.); 2 School of Sport, Exercise and Health Sciences, Loughborough University, Loughbororugh LE11 3TU, UK; L.James@lboro.ac.uk; 3 Institute of Cellular Medicine, Human Nutrition Research Centre, William Leech Building, Medical School, Newcastle University, Newcastle upon Tyne NE2 4HH, UK; emma.stevenson@newcastle.ac.uk

**Keywords:** females, dairy, energy intake, subjective appetite, cycling exercise

## Abstract

This study was designed to assess the effect of a dairy-based recovery beverage on post-exercise appetite and energy intake in active females. Thirteen active females completed three trials in a crossover design. Participants completed 60 min of cycling at 65% *V̇*O_2peak_, before a 120 min recovery period. On completion of cycling, participants consumed a commercially available dairy-based beverage (DBB), a commercially available carbohydrate beverage (CHO), or a water control (H_2_O). Non-esterified fatty acids, glucose, and appetite-related peptides alongside measures of subjective appetite were sampled at baseline and at 30 min intervals during recovery. At 120 min, energy intake was assessed in the laboratory by *ad libitum* assessment, and in the free-living environment by weighed food record for the remainder of the study day. Energy intake at the *ad libitum* lunch was lower after DBB compared to H_2_O (4.43 ± 0.20, 5.58 ± 0.41 MJ, respectively; *p* = 0.046; (95% CI: −2.28, −0.20 MJ)), but was not different to CHO (5.21 ± 0.46 MJ), with no difference between trials thereafter. Insulin and GLP-1_7-36_ were higher following DBB compared to H_2_O (*p* = 0.015 and *p* = 0.001, respectively) but not to CHO (*p* = 1.00 and *p* = 0.146, respectively). In addition, glucagon was higher following DBB compared to CHO (*p* = 0.008) but not to H_2_O (*p* = 0.074). The results demonstrate that where DBB consumption may manifest in accelerated recovery, this may be possible without significantly affecting total energy intake and subsequent appetite-related responses relative to a CHO beverage.

## 1. Introduction

In recent years, interest surrounding the role of dairy-based beverages (DBB) as post-exercise recovery aids has increased [1]. Research observing the effects of post-exercise DBB consumption have identified enhanced muscle protein synthesis [2], reduced exercise-induced muscle damage [3,4,5,6,7], and increased rehydration [8], thus accelerating recovery and subsequent performance [9]. Based on the available literature, it appears that many of these effects are a product of the nutritional composition of DBB which comprises high-quality proteins, low glycemic carbohydrates, in addition to electrolytes, vitamins, and minerals [10]. Dairy proteins, specifically casein and whey, constitute approximately 80% and 20%, respectively, of the total protein in milk, and provide an abundance of essential amino acids [11,12]. These amino acids are necessary to increase protein turnover and may subsequently act to repair and remodel damaged tissues following exercise. In addition to muscle tissue repair, the carbohydrate present in DBB (combined with dairy proteins) act to replace depleted glycogen stores [13], and the mixture of nutrients (again combined with dairy proteins) may enhance post-exercise rehydration compared to the ingestion of a carbohydrate-electrolyte beverage or water [8,14].

Outside of the recovery-related benefits, an emergent body of evidence supports the hypothesis that dairy-based foods elicit anti-obesity properties, providing a modest protective effect against adiposity [15,16]. To date, efforts to establish the underlying relationship between dairy consumption and adiposity have identified several putative mechanisms. Evidence from adult studies indicates that the consumption of dairy may inhibit lipid accretion and influence adipocyte lipid metabolism [17]. Furthermore, dairy consumption may act to potentiate several anorexigenic hormonal peptides of gastrointestinal, pancreatic, and adipose tissue origin that might influence appetite regulation compared to energy-equivalent products [18,19,20,21], improving subjective satiety and reducing energy intake [22,23]. Consequently, it has been suggested that dairy-based foods contribute to body mass regulation and, thus, energy balance, through actions on appetite and eating behaviour [24]. Interestingly, the same may stand true for exercise [25,26]. Certainly, regular exercise is often cited as a method for body mass maintenance and weight loss purposes. Taken together, it could be argued that dairy-based foods/beverages may enhance post-exercise recovery, whilst also beneficially modulating energy balance to facilitate body mass maintenance and/or weight loss. Many individuals, particularly women, exercise on a regular basis for weight loss or weight maintenance purposes [27] and, therefore, a DBB post-exercise may be an ideal drink to consume.

Presently, little information exists on the effects of post-exercise DBB consumption on subsequent appetite-related responses, especially in females. This is surprising given that both exercise and dairy-based foods/beverages may exert influence on appetite and eating behavior. The authors have previously demonstrated that post-exercise skimmed milk consumption reduces energy intake in recreationally-active females relative to an energy and volume matched serving of fruit-juice [28]. While our preliminary findings provide promise, in this study we observed no effect on measures of subjective appetite. Furthermore, the authors failed to quantify appetite-related peptides which may have provided valuable insights concerning the mechanisms impacting on appetite and energy intake. Consequently, there is a need to better understand the behavioral and physiological effects of exercise and post-exercise DBB consumption. Therefore, the aim of this study was to investigate the effect of a DBB following moderate intensity exercise on subsequent appetite and energy intake in recreationally active females.

## 2. Materials and Methods

### 2.1. Experimental Design

This study was a repeated measures, within-subject crossover study. The counterbalance randomization process was performed by one member of the research team and completed with the use of a web-based randomization tool [29]. Participants attended the nutrition and metabolism laboratories at the University of Northumbria on four occasions. The first visit served as a familiarization session and preliminary testing also took place. During the next three visits participants performed 60 min of cycling, before a 120 min recovery period, with all exercise carried out using a cycle ergometer (Monarch Weight Ergometer 839 E, Varberg, Sweden), followed by one of three randomly assigned recovery beverages. Samples of antecubital-venous blood and subjective measures of appetite were collected at baseline and at 30 min intervals for 120 min during recovery.

### 2.2. Participants

Thirteen recreationally-active females (mean ± SD, age 23 ± 4 years, body mass 63.5 ± 9.0 kg, stature 165.7 ± 6.3 cm, BMI 23.1 ± 2.9 kg·m**^−^**^2^, *V̇*O_2peak_ 43.5 ± 11.6 mL·kg·min**^−^**^1^) agreed to participate in this study. The study was conducted according to the guidelines laid down in the 2013 Declaration of Helsinki, and all experimental procedures involving human participants were approved by the Faculty of Health and Life Sciences Ethical Committee of the University of Northumbria. Written informed consent was obtained from all participants prior to data collection. Participants completed a self-report menstrual cycle questionnaire to determine menstrual cycle phase and contraceptive use (all participants were using oral contraceptives). Subsequently, all testing took place during the follicular phase of menstruation (days 1–14) to remove the impact of hormone interaction on substrate utilization [30].

### 2.3. Visit 1

Seven days preceding data collection, all participants underwent a familiarization session. Participants were familiarized with the equipment and methodological procedures that were to be employed in the study. This included the subjective appetite visual analogue scales (VAS), blood sampling, gas collection equipment, and all test foods. Secondly, as recommended by Livingstone *et al.* [31] participants were trained and educated on the processes required to appropriately document free-living energy intake. In addition, anthropometric measures of stature (stretch stature technique; Seca, Birmingham, UK) and body mass (Seca, Birmingham, UK) were collected to the nearest 0.1 cm and 0.1 kg, respectively. Finally, participants completed a discontinuous exercise test using a cycle ergometer to determine peak oxygen consumption (*V̇*O_2peak_). The test comprised of 4 min increments separated with 2 min rest, during which workload increased until volitional exhaustion. From this, a work rate equivalent to participants’ 65% *V̇*O_2peak_ was established and used for subsequent visits. The *V̇*O_2peak_ protocol was completed at 70RPM. The first stage of the discontinuous test commenced at 70 watts (W), and the workload was subsequently increased by 35 W per stage until the participant reached volitional exhaustion.

### 2.4. Pre-Trial Standardization

For the 24 h preceding the first experimental trial participants recorded all food and fluid consumption using a self-reported, weighed food diary. Participants were also instructed to refrain from caffeine and alcohol consumption (≥12-h) and strenuous physical activity (≥24-h) preceding data collection periods. These dietary and exercise habits were replicated preceding subsequent trials.

On the morning of each experimental trial, participants consumed a pre-prepared standardized breakfast meal. This was consumed in the participants’ home at 0700 h, following at least a 10 h overnight fast. Breakfast consisted of semi-skimmed milk (Tesco, London, UK) and cereal (Kellogg’s Rice Krispies, Manchester, UK), provided to participants in a cereal to milk ratio of 30 g:125 mL. The quantity issued provided 10% of the participants estimated daily energy requirement (with 14%, 14%, and 72% of the energy in the breakfast derived from protein, fat and carbohydrate, respectively) as previously used and drawn from recommendations from the National Diet and Nutrition Survey [32]. Individual daily energy requirements were computed according to age- and sex-specific calculations [33], providing an estimate of basal metabolic rate. Estimated values of basal metabolic rate were further multiplied against a physical activity factor of 1.7. To ensure and monitor compliance, participants were requested to return empty breakfast containers.

### 2.5. Visit 2, 3, and 4

For other trials, participants arrived at the nutrition and metabolism laboratory at 0900 h. Upon waking and until arrival at the clinical testing laboratory the consumption of water only was permitted. Participants were requested to record, document, and replicate morning water consumption (if any) for subsequent trials. On arrival, participants were rested and an indwelling cannula was inserted into an antecubital vein for blood sampling. A pre-exercise (−60 min) blood sample was drawn and participants completed a series of pre-exercise subjective appetite VAS. Following pre-exercise measurements, participants completed 60 min continuous cycling exercise at a work rate that elicited 65% of each participants *V̇*O_2peak_. During exercise, samples of expired air, heart rate, and ratings of perceived exertion (RPE) were obtained at regular intervals. On immediate termination of exercise, a recovery beverage was consumed (Table 1). Following consumption, participants remained at rest and completed a 120 min recovery period in an environment free from food cues, where further blood samples and VAS were collected every 30 min. At 120 min, a homogenous *ad libitum* pasta meal was provided. Participants were instructed to eat until comfortably full and satisfied, and were given 30 min to consume the meal. On completion of the *ad libitum* pasta meal participants were free to leave the laboratory. For the remainder of the study day, participants were requested to not engage in any type of activity and were asked to record any further food and drink intake using a weighed food diary.

### 2.6. Recovery Beverages

Participants were given 15 min to consume the entire contents of the beverage which included (1) a commercially available DBB (nouriSH me now™, Sheffield, UK); (2) a 15% commercially available carbohydrate beverage ((CHO) Lucozade Energy Orange™, GlaxoSmithKline, London, UK); or (3) an energy-free water control (H_2_O). All beverages were matched for volume, and DBB and CHO matched for energy content. Post-exercise recovery beverages were distributed in a counterbalanced manner. Beverages were served chilled at 4 **°**C and in opaque water bottles.

### 2.7. Gas Analysis

To collect gas samples, a mouthpiece attached to a two-way, non-rebreathing valve (model 2730, Hans Rudolph, Kansas City, MO, USA) was used. Gas samples, collected in Douglas Bags, were analysed for concentrations of oxygen and carbon dioxide using paramagnetic and infrared transducers, respectively (Service 5200S, Crowborough, Sussex, UK). In addition, bag volume and temperature of expired gas samples were determined using a dry gas meter (Harvard Apparatus, Edenbridge, Kent, UK) and thermistor (model 810-080, ETI, Worthing, UK), respectively. Expired gas samples (60 s) were collected at the end of every 10 min period (6 samples). From this the energy cost of exercise was estimated.

### 2.8. Subjective Appetite

Subjective measures of appetite were assessed using validated 100 mm, paper based VAS [34]. Scales were anchored with diametrically opposed feelings of extremity, and addressed hunger (“how hungry do you feel?”), gut fullness (“how full do you feel?”), prospective food consumption (“how much do you think you can eat?”), satisfaction (“how satisfied do you feel?”), and nausea (“how nauseous do you feel now?”). Participants were required to report their self-perceived appetite immediately prior to each blood sample (pre-exercise (−60), 30, 60, 90, and 120 min). Scales were issued in the same order at each sample point, and ratings measured by the same researcher to minimize discrepancies.

### 2.9. Blood Sampling and Analysis

At five separate intervals, antecubital-venous (4.0 mL) blood samples were drawn into pre-cooled EDTA-treated monovettes. Samples were collected at pre-exercise (−60) and at 30, 60, 90, and 120 min following recovery beverage consumption. Participants lay supine for approximately 5 min prior to each blood sample. Patency of the cannula was preserved by flushing a small volume of non-heparinized saline (0.9% NaCl; Becton, Dickinson and Company, Franklin Lakes, NJ, USA) through the connector tube on completion of each sample. Residual saline waste was discarded immediately before succeeding sample points, avoiding contamination and dilution of antecubital-venous blood.

Pre-analytical (e.g., sample treatment) and analytical (e.g., sample handling) procedures were followed in an identical manner to our previous studies [35,36]. Consequently, monovettes contained aprotinin (25 µL/mL whole blood) for the preservation of glucagon-like peptide 1_7-36_ (GLP-1_7-36_) and glucagon. On collection, samples were placed on ice and centrifuged at 1509× *g* (3000 rpm) for 10 min at 4 °C within 5 min of collection. Aliquots of plasma supernatant were stored immediately at −80 °C for later determination of glucose, non-esterified fatty acids (NEFA), GLP-1_7-36_, glucagon, insulin, and leptin. From the aliquots, a 20 µL capillary tube was filled with plasma to determine glucose using an automated glucose analyser by the glucose oxidase method (Biosen C_Line, EKF Diagnostics, Cardiff, UK). Concentrations of NEFA (mmol/L) were determined using enzymatic colorimetric assays (Randox Laboratories, County Antrim, UK). Quantitative assessments of GLP-1_7-36_ (pmol/L), glucagon (pg/mL), insulin (pmol/L), and plasma leptin (ng/mL) were simultaneously determined in 40 µL of plasma by electrochemiluminescence using a human hormone multiplex assay kit (Sector Imager 2400, MesoScale Discovery, Rockville, MD, USA). Of note, the addition of protease inhibitors to samples for the preservation of GLP-1_7-36_ and glucagon does not influence measured concentrations of plasma leptin and insulin [37]. Each participant’s samples were analyzed on the same plate to minimize variation. Intra-assay CVs were <7% for all biochemical analysis except for GLP-1_7-36_, which was 12%.

### 2.10. Energy Intake Assessment

Energy intake was assessed at two occasions. Firstly, lunchtime food intake (120 min) was assessed through *ad libitum* intake of a homogenous pasta meal. The meal comprised of pasta (Tesco, London, UK), tomato sauce (Tesco, London, UK), cheddar cheese (Tesco, London, UK), and olive oil (Tesco, London, UK). Detailed information concerning the nutrient composition of the pasta meal and the method of cooking has been reported previously [35]. The *ad libitum* test meal was consumed in isolation in a room free from social influences. Participants were initially provided with a sub-serving of the whole portion, which was continuously replenished by the research team at regular intervals throughout consumption. Continuously replenishing the pasta meal ensures participants terminate eating when comfortably full, and not due to the cue of an empty bowl. Energy intake from the pasta meal was calculated based on the amount consumed and nutritional composition as indicated by the manufacturer. To facilitate this, research staff covertly weighed the meal prior to serving, and immediately following meal termination. Secondly, participants recorded all food and drink items consumed for the remainder of each trial day, utilizing self-reported weighed records to document food intake. One member of the research team examined all food records utilizing the nutritional software package Nutritics (Nutritics Professional v3.09, Nutritics, Dublin, Ireland).

### 2.11. Statistical Analysis

All data are presented as mean ± standard error of the mean (SEM). Statistical software package (IBM SPSS v22, Armonk, NY, USA) was used for inferential analysis and significance was accepted at the *p* < 0.05 *a priori*. For data with multiple time-points (blood analyte concentrations and subjective appetite sensations), two-way (trial, 3 × time, 5) repeated measures analysis of variance (ANOVA) was performed to assess for differences in trial and time. Blood analyte concentrations and subjective appetite sensations during the 120 min recovery period (time points: 30, 60, 90, and 120 min) were also computed as time-averaged (90 min) concentrations using the trapezoidal rule [38]. Time-averaged concentrations for these variables during each trial were analyzed using one-way repeated measures ANOVA. Due to difficulties associated with blood sampling, where data for a single time point was missing (14 time points were missing out of a total 190 (<8%)) for each blood-based variable, linear interpolation was used to complete the data set. In addition, due to difficulties in data collection, subjective appetite data is presented for 10 participants.

One-way repeated measures ANOVAs were also used to detect differences between trials in exercise energy expenditure (MJ), absolute and relative energy intake at the test meal (MJ), and total energy intake (MJ). Absolute energy intake (MJ) was considered as the absolute amount of pasta consumed at the *ad libitum* test meal. Relative energy intake (MJ) (accounting for the energy content of the recovery beverages and energy cost during the cycling exercise) was determined by totalling absolute energy intake at the pasta meal with the energy content of the beverages and subtracting the energy cost of exercise (MJ). Total energy intake was determined as the sum of breakfast, recovery beverage, *ad libitum* test meal and free-living energy intake. Data were checked for normal distribution with the use of the Kolmogorov-Smirnov normality test and were log-transformed if appropriate before statistical analysis. Mauchley’s test assessed the sphericity of the data and where appropriate, violations were corrected using the Greenhouse-Geisser estimate. Where significant effects occurred, *post-hoc* analyses were Bonferroni-adjusted paired *t*-tests. Unless otherwise stated, 95% confidence intervals (95% CI) are presented for mean differences between trials.

## 3. Results

### 3.1. Exercise Measurements

Gross energy cost during the 60 min cycling exercise was 2.64 ± 0.26 MJ (DBB), 2.69 ± 0.29 MJ (CHO), and 2.59 ± 0.24 MJ (H_2_O) and was not different between trials (*p* = 0.409; (grand mean 95% CI: 2.07, 3.21)). Sessional RPEs were not significantly different between trials (*p* = 0.657; (grand mean of 12 ± 1 and 95% CI: 11, 13)). Similarly the mean HR during the 60 min exercise was not different between trials (*p* = 0.326; (grand mean of 155 ± 4 and 95% CI: 146, 164 bpm)).

### 3.2. Energy Intake

**Figure 1:**
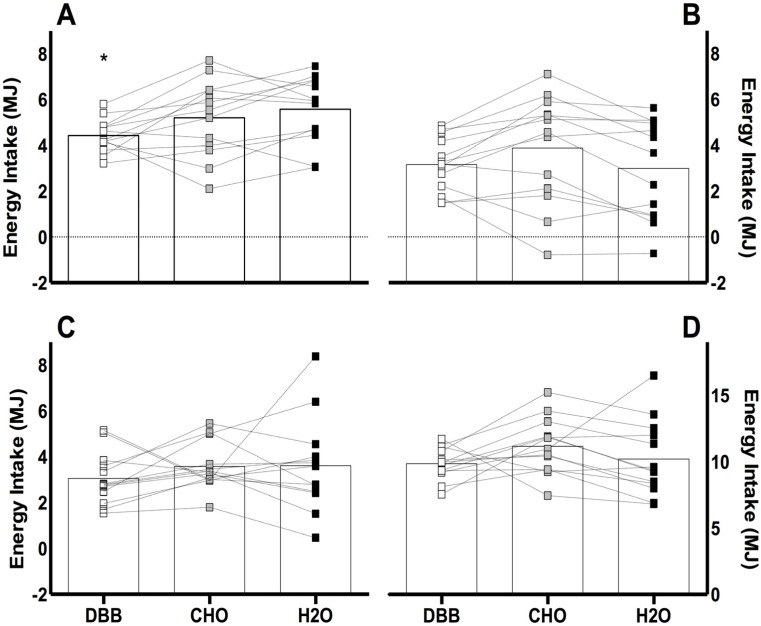
Mean and individual responses for absolute (**A**, *n* = 13), and relative (**B**, *n* = 13) energy intake at the *ad libitum* meal, free-living (**C**, *n* = 13) and total (**D**, *n* = 13) energy intake (MJ). Values are presented as mean alongside individual responses. Significance at the *p* < 0.05 level. * denotes a significant difference compared to H_2_O.

Energy intake at the *ad libitum* pasta meal (Figure 1) was lower following DBB compared to H_2_O (4.43 ± 0.20, 5.58 ± 0.41 MJ, respectively; *p* = 0.046; (95% CI: −2.28, −0.20)), but was no different to CHO (5.21 ± 0.46 MJ *p* = 0.211; (95% CI: −1.88, 0.31)). Further analysis revealed this was not influenced by trial order (*p* = 0.164). No statistical differences were found between trials for relative energy intake, free-living energy intake or total energy intake. At the *ad libitum* meal, the Delta (Δ) relative energy intake between trials were 0.16 ± 0.38, 0.89 ± 0.29, and −0.73 ± 0.42 MJ for DBB-H_2_O, CHO-H_2_O, and DBB-CHO, respectively. For total daily energy intake, Delta (Δ) values between trials were −0.34 ± 0.89, 0.95 ± 0.61, and −1.29 ± 0.67 MJ for DBB-H_2_O, CHO-H_2_O, and DBB-CHO, respectively.

### 3.3. Subjective Appetite Responses

Analysis revealed differences across all trials for time-averaged subjective appetite measures, except for nausea (Table 2). Relative to H_2_O control, DBB, and CHO enhanced subjective fullness (*p* = 0.003; (95% CI: 10.8, 44.9 mm), and *p* = 0.021; (95% CI: 2.0, 24.5 mm), respectively), and satisfaction (*p* = 0.014; (95% CI: 5.3, 44.0 mm), and *p* = 0.012; (95% CI: 3.8, 30.1 mm), respectively). Consistent with this, subjective hunger was lower following DBB and CHO compared to H_2_O control (*p* = 0.032; (95% CI: −37.9, −1.7 mm), and *p* = 0.019; (95% CI: −18.5, −1.8 mm), respectively). In addition, DBB reduced prospective food consumption (*p* = 0.008; (95% CI: −31.5, −5.4 mm)) compared to H_2_O. Analysis revealed main effects of time and trial for all subjective appetite measures. Time × trial interaction (*p* = 0.005) showed that satisfaction was reduced at 30 and 60 min following H_2_O control relative to DBB (*p* = 0.019; (95% CI: −62.8, −5.8 mm), and *p* = 0.009; (95% CI: −53.8, −8.6 mm), respectively) and CHO (*p* = 0.032; (95% CI: −37.9, −1.7 mm), and *p* = 0.004; (95% CI: −34.2, −7.2 mm), respectively). At 120 min, satisfaction following CHO consumption was greater than that following H_2_O (*p* = 0.036; (95% CI: 0.6, 17.6 mm)).

### 3.4. Blood Parameters

#### 3.4.1. Plasma Glucose

**Figure 2:**
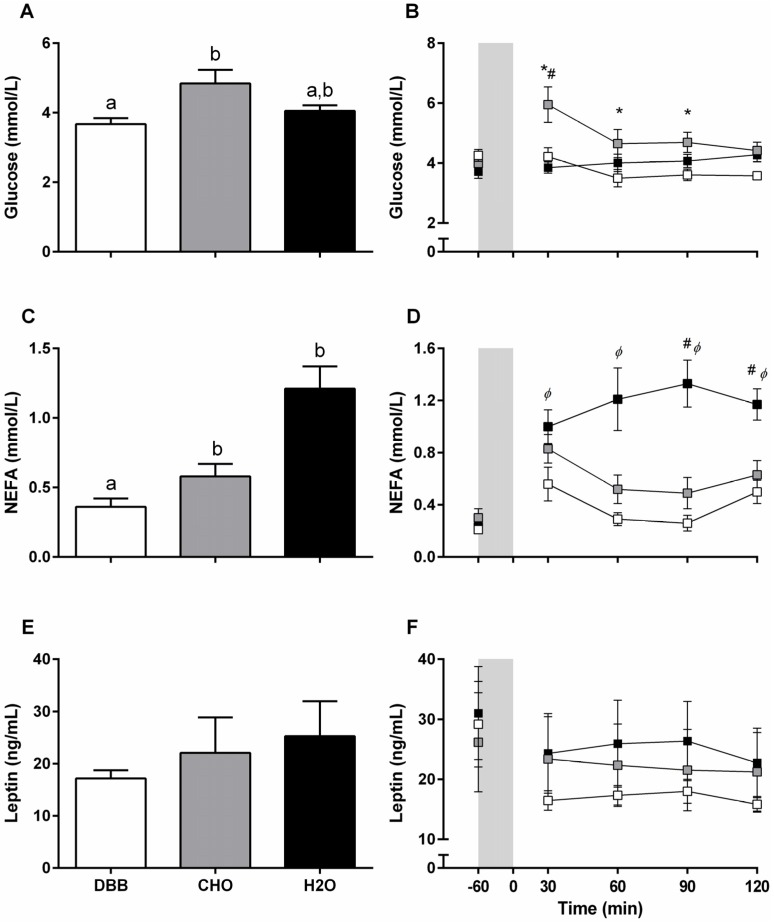
Time-average (90 min) (**A**,**C**,**E**) and plasma concentrations (**B**,**D**,**F**) of glucose (mmol/L), NEFA (mmol/L), and leptin (ng/mL), respectively, pre (−60) and throughout the 120 min recovery period (*n* = 13). Vertical grey shaded area (**B**,**D**,**F**) represent the 60 min cycling bout. Values are presented as mean ± SEM. Values with unlike letters (^a,b^) denote significant difference between trials. White filled boxes (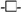) represent DBB, grey filled boxes (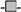) represent CHO, and black filled boxes (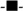) represent H_2_O. Significance at the *p* < 0.05 level. *ϕ* denotes significant difference between DBB and H_2_O; ^#^ denotes significant difference between CHO and H_2_O; * denotes significant difference between DBB and CHO.

Time-averaged glucose was higher following CHO compared to DBB (4.84 ± 0.39, 3.67 ± 0.17 mmol/L, respectively; *p* = 0.001; (95% CI: 0.38, 1.96)) but not to H_2_O (4.05 ± 0.16 mmol/L; *p* = 0.169; (95% CI: −0.16, 1.75)). There was no difference between DBB and H_2_O (*p* = 0.355; (95% CI: −0.96, 0.22)) (Figure 2A). A time × trial interaction (*p* < 0.001) revealed that the increase in glucose at 30 min post beverage ingestion was highest in CHO compared to DBB and H_2_O control (*p* = 0.023; (95% CI: 0.23, 3.23), and *p* = 0.007; (95% CI: 0.58, 3.61) respectively), and remained elevated compared to DBB at 60 and 90 min post beverage consumption (*p* = 0.015; (95% CI: 0.22, 2.01), and *p* = 0.007; (95% CI: 0.29, 1.67), respectively) (Figure 2B).

#### 3.4.2. Plasma NEFA

Time-averaged NEFA was lower following DBB (0.36 ± 0.06 mmol/L) compared to CHO (0.58 ± 0.09 mmol/L; *p* = 0.039; (95% CI: −0.47, 0.03)) and H_2_O (1.21 ± 0.16 mmol/L; *p* < 0.001; (95% CI: −1.33, −0.37)). There was no difference between CHO and H_2_O (*p* = 0.068; (95% CI: −1.25, −0.01)) (Figure 2C). Main effects for time and trial were observed following two-way repeated measures ANOVA. A time × trial interaction (*p* = 0.001) revealed that increases in NEFA at 30 min post beverage consumption were greater in H_2_O compared to DBB (*p* = 0.042; (95% CI: 0.01, 0.61)). Plasma NEFA concentrations 60 min post beverage consumption were reduced in DBB (*p* = 0.001; (95% CI: −0.93, −0.25)), and for both DBB and CHO at 90 min (*p* < 0.001; (95% CI: −1.04, −0.43), and *p* = 0.028; (95% CI: −0.97, −0.05), respectively) and 120 min (*p* < 0.001; (95% CI: −0.62, −0.22), and *p* = 0.030; (95% CI: −0.62, −0.03), respectively) post beverage consumption compared to H_2_O (Figure 2D).

#### 3.4.3. Plasma Leptin

No differences in time-averaged leptin were evident between test beverages (17.17 ± 1.59, 22.08 ± 6.79, 25.30 ± 6.67 ng/mL for DBB, CHO and H_2_O, respectively; *p* = 0.289; (grand mean 95% CI: 11.42, 31.62)) (Figure 2E) and two-way anova demonstrated main effect of time across trials only (Figure 2F).

#### 3.4.4. Plasma Insulin

**Figure 3:**
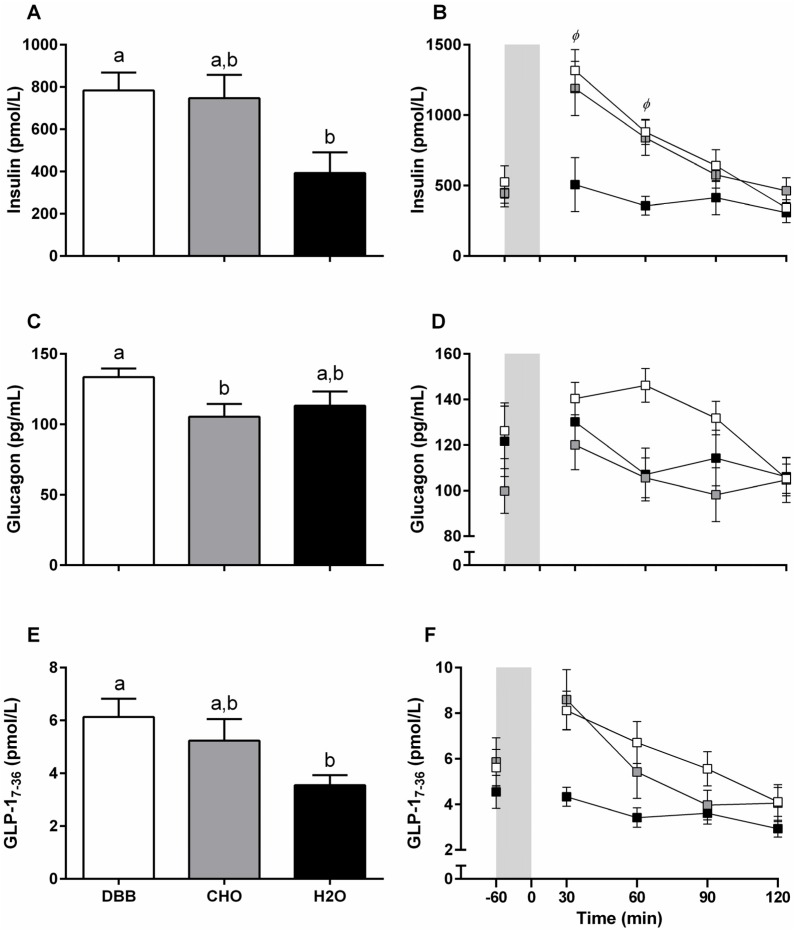
Time-average (90 min) (**A**,**C**,**E**) and absolute postprandial plasma concentrations (**B**,**D**,**F**) of insulin (pmol/L), glucagon (pg/mL), and GLP-1_7-36_ (pmol/L) respectively pre (−60) and throughout the 120 min recovery period (*n* = 13); Vertical grey shaded area (**B**,**D**,**F**) represent the 60 min cycling bout. Values are presented as mean ± SEM. Values with unlike letters (^a,b^) denote significant difference between trials. White filled boxes (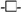) represent DBB; grey filled boxes (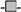) represent CHO; and black filled boxes (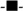) represent H_2_O. Significance at the *p* < 0.05 level. *ϕ* denotes significant difference between DBB and H_2_O.

Time-averaged insulin was higher following consumption of DBB compared to H_2_O (783.85 ± 83.96, 392.58 ± 98.14 pmol/L respectively; *p* = 0.015; (95% CI: 69.18, 713.36)) but not to CHO (747.3 ± 109.73 pmol/L; *p* = 1.00; (95% CI: −270.02, 343.11)). There was no difference between CHO and H_2_O (*p* = 0.152; (95% CI: −136.94, 846.39) (Figure 3A). Main effects for time and trial were observed following two-way repeated measures ANOVA. A time × trial interaction (*p* < 0.001) revealed that the increase in insulin was greater in DBB compared to H_2_O at 30 min (*p* = 0.001; (95% CI: 0.27, 0.96)) and 60 min (*p* < 0.001; (95% CI: 0.22, 0.69)) post beverage ingestion (Figure 3B).

#### 3.4.5. Plasma Glucagon

Time-averaged glucagon was higher following DBB compared to CHO (133.6 ± 6.1, 105.4 ± 9.0 pg/mL respectively; *p* = 0.008; (95% CI: 5.3, 51.1)) but not to H_2_O (113.2 ± 10.1 pg/mL; *p* = 0.074; (95% CI: −9.7, 50.6)). There was no difference between CHO and H_2_O (*p* = 0.911; (95% CI: −28.4, 12.9) (Figure 3C). While main effects for time and trial were observed, no interaction effects were present (Figure 3D).

#### 3.4.6. Plasma GLP-1_7-36_

Time-averaged GLP-1_7-36_ was higher following DBB compared to H_2_O (6.13 ± 0.69, 3.55 ± 0.38 pmol/L respectively; *p* = 0.001; (95% CI: 0.79, 4.36)) but not to CHO (5.24 ± 0.81 pmol/L; *p* = 0.146; (95% CI: −0.63, 2.41)). There was no difference between CHO and H_2_O (*p* = 0.249; (95% CI: −0.53, 3.91) (Figure 3E). Two-way ANOVA demonstrated a main effect of time across trials and a main effect of trial (Figure 3F).

## 4. Discussion

To our knowledge, while our previous work has examined the effect of post-exercise DBB consumption on subsequent appetite and energy intake in active females [28], this is the first study where measures of relevant hormonal appetite-related peptides were also quantified. The main findings arising from this study are that post-exercise DBB consumption reduced energy intake at the *ad libitum* meal by 23% (1.14 MJ; *p* = 0.046), and 16% (0.78 MJ; *p* = 0.211) compared to H_2_O and CHO consumption, respectively, while subsequent appetite (subjective and hormonal appetite-related peptides) and energy intake was similar between the commercially available beverages. In addition, although significance was not obtained, DBB reduced overall energy intake by −1.29 ± 0.67 MJ compared to a volume and energy matched CHO beverage. It is recognized that post-exercise DBB consumption may enhance muscle protein synthesis [2], attenuate exercise-induced muscle damage [3,4,5,6,7], and increase rehydration [8], thus manifesting in accelerated recovery and improved performance [9]. Consequently, these data may begin to illustrate an additional role for DBB consumption in the immediate post-exercise period for individuals exercising for weight loss/maintenance purposes, though further research is warranted to substantiate these initial findings.

The results concerning energy intake at the *ad libitum* meal are concordant with a previous study investigating the effects of post-exercise beverage macro-nutrient content on appetite and energy intake [39]. Clayton and colleagues [39] found that *ad libitum* energy intake (following 30 min cycling exercise and 60 min after drink ingestion) was lower following a 6% whey protein isolate solution compared to a placebo, but was not different after an isoenergetic carbohydrate beverage. Early research suggests that the energy content of a beverage is the only property that significantly affects energy intake at a subsequent meal as opposed to nutritional composition [40]. Consequently, considering the recovery beverages used in this study were matched for energy content it may be unsurprising that energy intake at a subsequent *ad libitum* meal was similar between DBB and CHO. Interestingly, at the *ad libitum* meal, 10 of 13 participants consumed less energy following DBB when compared to both H_2_O and CHO. However as expected, when the energy content of the beverages were considered, H_2_O elicited the greatest reduction in relative energy intake at the *ad libitum* meal as demonstrated by the delta (Δ) values; 0.16 ± 0.38, 0.89 ± 0.29, and −0.73 ± 0.42 MJ for DBB-H_2_O, CHO-H_2_O and DBB-CHO, respectively. Similarly, when all food intake was considered, total daily energy intake was not significantly different between DBB (9.86 ± 0.33 MJ) compared to H_2_O (10.20 ± 0.79 MJ) and CHO (11.15 ± 0.58 MJ). These findings are in agreement with the recent study by Clayton *et al.* [39]. However, when delta (Δ) between trials for total daily energy intake is considered, DBB delivered the greatest reduction in total daily energy intake compared to other trials (−0.34 ± 0.89, 0.95 ± 0.61 and −1.29 ± 0.67 MJ for DBB-H_2_O, CHO-H_2_O ,and DBB-CHO, respectively). Indeed, 11 of 13 participants consumed less total daily energy following DBB compared to CHO and six compared to H_2_O. It has been suggested in the scientific literature that daily deficits in energy intake of 0.71 MJ (712 kJ, 170 kcal) may be “clinically” meaningful from a weight loss and/or maintenance perspective [41]. Based on our findings, one may speculate that post-exercise DBB and/or H_2_O consumption shows some promise as a potential application for weight maintenance or loss compared to a volume and energy matched CHO beverage, although the added nutritional benefit of DBB must be considered. Nevertheless, further research is certainly warranted to establish whether longer-term post-exercise DBB consumption serves as a beverage to aid weight maintenance and weight loss.

Relative to carbohydrate-based beverages and water, dairy beverages contribute significantly to the consumption of high-quality proteins. It is suggested that dietary proteins are more satiating than energetic equivalents of carbohydrate or fat under most conditions, and suppress short-term energy intake at the next available opportunity [19,42,43]. In support of this, the effect of high protein food on short-term food intake reflects subjective measurements of appetite [19]. Foods high in protein not only suppress short-term food intake to a greater extent than carbohydrate and fat but also provide strong feelings of satiety after their consumption [43,44]. In this study, consumption of DBB and CHO recovery beverages elicited similar responses on subjective appetite compared with H_2_O, and may suggest that the energy content of a beverage remains the primary characteristic influencing subsequent appetite responses [39]. It is important to note, however, that alterations in subjective perceptions of food-related emotions do not always translate and reflect actual eating behaviour [45]. In our previous study, for example, significant reductions in relative energy intake were observed 60 min following 30 min of cycling, yet did not impact on subjective appetite [28]. Furthermore, reductions in subsequent energy intake and appetite responses have principally been observed after consuming beverages containing large amounts (30 + g) of protein [28,46]. It is probable that our study was underpowered to detect differences in energy intake deemed clinically meaningful [41] following DBB consumption. In addition, we may not have observed a sufficiently large reduction in energy intake following DBB consumption due to our methodological approach (e.g., insufficient protein content of the beverages, exercise duration, and time lapse between exercise and consumption of the test meal).

In an attempt to establish the physiological effects of exercise and post-exercise dairy beverage consumption, we measured hormonal peptides of gastrointestinal, pancreatic, and adipose tissue origin that are implicated in appetite regulation and metabolism. Importantly, a growing body of evidence suggests that an acute bout of aerobic exercise (perhaps more so than resistance exercise) influences appetite-related hormones (for review please refer to [47]). While exercise may confer only transient changes in circulating hormones, the suppression offered by the exercise itself may have contributed to the appetite responses and subsequent energy intake that was observed in the present investigation. Having said this, given that the acute exercise bout employed was repeated across trials, the contribution of exercise would have been analogous. Consequently, any differences between trials were likely due to the recovery beverage consumed. The postprandial response of these peptides is profoundly influenced by energy consumed as well as the distribution of ingested macro- (and micro-) nutrients. For example, circulating concentrations of GLP-1_7-36_ increase immediately following food consumption in direct proportion to energy content [48], but similarly when carbohydrates and fats are present [49]. Postprandial concentrations of glucagon are more capricious, but rise following fasting, protein ingestion [50] or exercise [51]. It may therefore be unsurprising that post-exercise DBB and CHO consumption resulted in a greater plasma GLP-1_7-36_, considering both beverages were isoenergetic (1.36 MJ). Furthermore, the elevated postprandial glucagon and insulin concentrations following DBB consumption may have been predictable due to its protein content. Although several studies have demonstrated glucagon, GLP-1_7-36_ and insulin to potently increase satiety and acutely reduce food intake in humans [52,53,54], the findings from this study do not fully support this. Together, the differences in subjective appetite and appetite-related peptides may have been insufficient to stimulate larger differences in subsequent energy intake; namely when comparing CHO and H_2_O, and DBB and CHO at the *ad libitum* meal, and energy intake beyond the first meal. Outside of the effects of protein on satiety, it is well established that protein elicits a greater effect on diet induced thermogenesis (20%–35% of energy consumed) compared to energy matched intakes of carbohydrate (5%–15% of energy consumed) or fat (0%–3% of energy consumed) [55]. Indeed, early investigations have reported an increased thermogenic effect after consumption of a DBB in comparison to an energy matched sugary beverage [56]. Additionally, there is evidence suggesting that glucagon [57,58] and calcium exerts properties that may stimulate energy expenditure and lipid utilization [58]. While not directly measured in the present investigation, collectively, this suggests that post-exercise DBB consumption may have further impacted on energy balance through an increase in energy expenditure and, perhaps, through altered substrate utilization.

Though the work presented throughout this manuscript has numerous strengths, the findings are not without limitation and warrant mention. Firstly, the results arising from this manuscript are drawn from an acute intervention. Consequently, observations require careful interpretation, as study findings may not translate over extended periods. In addition, the present findings are limited by the relatively small population sample of healthy recreationally-active females and, thus, the relevance to a wider population needs further evaluation. Nonetheless, research concerning the consumption of DBB on appetite and postprandial hormonal response is sparse, particularly in females. The observations arising from this study are therefore novel and present important information about post-exercise beverage consumption for females, especially as differences in energy-regulating hormones following exercise have been reported between men and women [59]. In turn, participants’ preconceived attitudes concerning the recovery beverages may have confounded any observations. While our study design provided robust experimental control, the provision of a limited meal (cheese and tomato pasta) and a pre-determined volume of the recovery beverage may not truly reflect actual free-living behaviours, especially considering this was repeated over three trials. Indeed, factors associated with palatability and gastrointestinal discomfort may have induced an element of progressive dislike that (if faced with) may have influenced subsequent eating behaviour and, thus, satiety. 

## 5. Conclusions

Considered together nonetheless, the present study demonstrates that the consumption of a DBB beverage immediately following exercise reduces overall energy intake by approximately −1.29 ± 0.67 MJ, compared to a volume and energy matched CHO beverage, despite eliciting similar responses concerning subsequent appetite and hormonal peptide responses. Consequently, the influence of post-exercise DBB consumption on subsequent appetite and energy intake remain unclear. Despite this, it is important to consider that DBB represent a nutrient-dense foodstuff and encompass an array of nutrients that confer numerous health benefits and may enhance post-exercise recovery, while contributing significantly to the consumption of high-quality nutrients and numerous bioactive constituents. Further research is warranted to fully elucidate the mechanisms influencing subsequent appetite and energy intake suppression in response to DBB and exercise, both alone and in combination.

## Figures and Tables

**Table 1:** Nutritional composition of the recovery beverages.

Nutritional Composition	DBB	CHO	H_2_O
Serving size (+mL water)	500 (24)	524	524
Energy (MJ)	1.36	1.36	0
Energy (kcal)	325	325	0
Carbohydrate (g)	56.7	78.6	0
Fat (g)	1.1	0	0
Protein (g)	22.2	<0.5	0

Abbreviations: DBB, Dairy-based beverage; CHO, carbohydrate beverage; H_2_O, water control.

**Table 2:** Time-averaged concentrations in the 2 h postprandial recovery period for all subjective appetite sensations (mm), *n* = 10, mean ± SEM.

Subjective Sensation	DBB	CHO	H_2_O
Hunger	63 ± 8 ^a^	72 ± 4 ^a^	83 ± 4 ^b^
Fullness	42 ± 6 ^a^	27 ± 5 ^a^	14 ± 4 ^b^
Satisfaction	37 ± 6 ^a^	29 ± 5 ^a^	12 ± 3 ^b^
Prospective Food Consumption	65 ± 3 ^a^	76 ± 4 ^a,b^	84 ± 3 ^b^
Nausea	13 ± 5	17 ± 8	9 ± 5

Abbreviations: DBB, dairy-based beverage; CHO, carbohydrate beverage; H_2_O, water control. ^a,b^ Values with unlike letters denote significant difference between trials. Significance at the *p* < 0.05 level.

## Abbreviations

The following abbreviations have been used throughout this manuscript.

CHO	Carbohydrate beverage
CI	Confidence interval
DBB	Dairy-based beverage
GLP-1_7-36_	Glucagon-like peptide-1
H_2_O	Water control
NEFA	Non-esterified fatty acids
VAS	Visual analogue scale
